# Supplementary material for: Analysis of Therapeutic Decisions for Infantile Hemangiomas: A Prospective Study Comparing the Hemangioma Severity Scale with the Infantile Hemangioma Referral Score
Source: Children (Basel). 2022 Nov 28;9(12):1851. doi: 10.3390/children9121851 (PMC9777170; doi:10.3390/children9121851)
Supplement: Supplementary file 1 [file children-09-01851-s001.zip › children-2048265-supplementary.pdf]

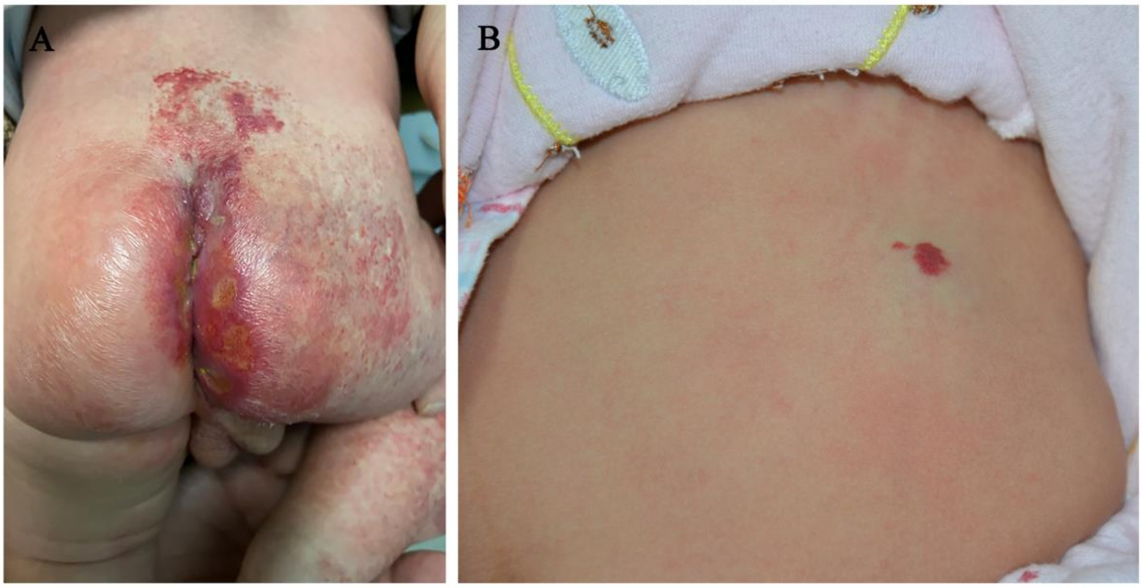

Supplementary Figure S1: A hip segmental IH with ulceration in a 10-month-old male patient. He was included in the treatment group. His HSS score was 16 points. He required referral after the HSS and IHReS assessments(A). A 6-month-old female patient with left abdomen IH was included in the non-treatment group. Her HSS score was 3 points. She was not need referral after the HSS and IHReS assessments(B).
